# Bayes or not Bayes, is this the question?

**DOI:** 10.3325/cmj.2019.60.50

**Published:** 2019-02

**Authors:** Branimir K. Hackenberger

**Figure 1:**
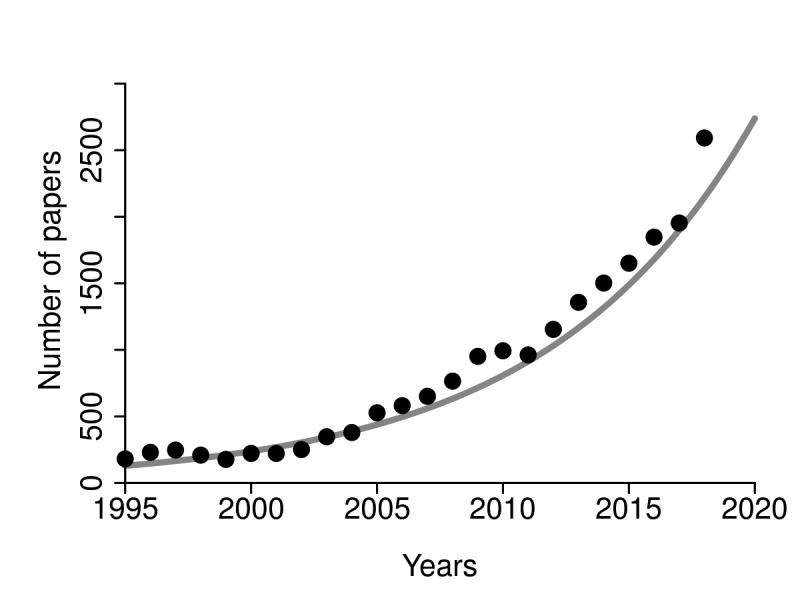
The number of published medical articles using Bayesian statistics in the period from 1995 to 2018 (sciencedirect.com, February 2019).

According to different scientific literature databases (eg, ScienceDirect, Web of Science), each year more and more scientific articles use Bayesian methods for data processing (Figure 1). Does this mean that Bayesian statistics is better than frequentist statistics? What can we achieve with Bayesian methods but not with frequentist methods? Will the use of Bayesian statistics add value to our research? What do I lose if I do not use it? Could the use of Bayesian statistics increase the potential for important medical discoveries? Is Bayesian statistics complicated?

The basis of Bayesian statistics is Bayes' theorem, which describes the probability of an event occurrence based on previous knowledge of the conditions associated with this event. For example, if the patient has difficulty remembering recent events and has mood swings and loss of motivation, how likely can we suspect Alzheimer disease? It is not easy to answer just on the basis of these symptoms. Furthermore, it makes a big difference if this patient is 16 years old or 75 years old. The age information strongly changes the likelihood that these symptoms occurred due to Alzheimer disease. In this easily understandable and intuitive example, Alzheimer disease is an event and age is a condition associated with this event.

The best example for explaining Bayesian statistics may be diagnostic tests. If we want to calculate the likelihood that one positively tested patient has the disease, one must know different expectations. First, we need to know the accuracy of the testing method. And second, we need to know the occurrence of the disease in the population. If we know that the accuracy of the test is 99% and that the disease appears in 1 out of 10 000 people, we can determine the probability that the positively tested patient is ill. One can intuitively conclude that this probability is 99%. However, this would be a mistake! The likelihood that the positively tested patient really has the disease in this case is less than 1%. Namely, the data on disease occurrence in the population, eg, prior probabilities, strongly influence the calculation. In this example, the appearance of disease is the prior probability, and the calculated probability of the illness of a positively tested person is the posterior probability. If the prior and posterior probabilities come from the same statistical distribution family, they are called conjugate distributions and the prior is named conjugate prior.

Bayesian statistics is older than frequentist statistics, but it has been neglected over the years. The main reason was the ability of Bayesian statistics to solve only a few cases when conjugate priors were known. Luckily, the development of information and computer technologies and the discovery of some new mathematical methods resurrected Bayesian statistics. Particularly notable was of the invention in the 1950s of Markov Chain Monte Carlo (MCMC) methods, such as the construction of random sampling algorithms from a probability distribution that enabled the calculation of Bayesian hierarchical models. A few years later, MCMC began to be used by statisticians, and the era of modern Bayesian statistics started. One of the earliest papers on the use of Bayesian statistics in medicine was published in 1982 (1).

In contrast to frequentist statistics, in Bayesian statistics all relevant information necessary to make an inference is contained in the observed data rather than in other unobserved quantities. Validity is maintained as long as the prior probability model is correctly specified regardless of prespecified experimental design.

One of the main objections against frequentist hypothesis testing is the use of *P* values, because the *P* value is partly determined by data that have never been observed. Bayesian methods use no null and alternative hypotheses, but in their case the main objection is that a prior is subjective. Moreover, there is no single, prescribed and well-defined method for choosing a prior. The consequence is that different people can use different priors for the same experiment and thus obtain different posteriors and make different conclusions. On the other hand, Bayesian methods only determine the probability of an event. Also, the possibility of using different priors allows the sensitivity of the experiment results to be measured for different priors.

**Figure 2:**
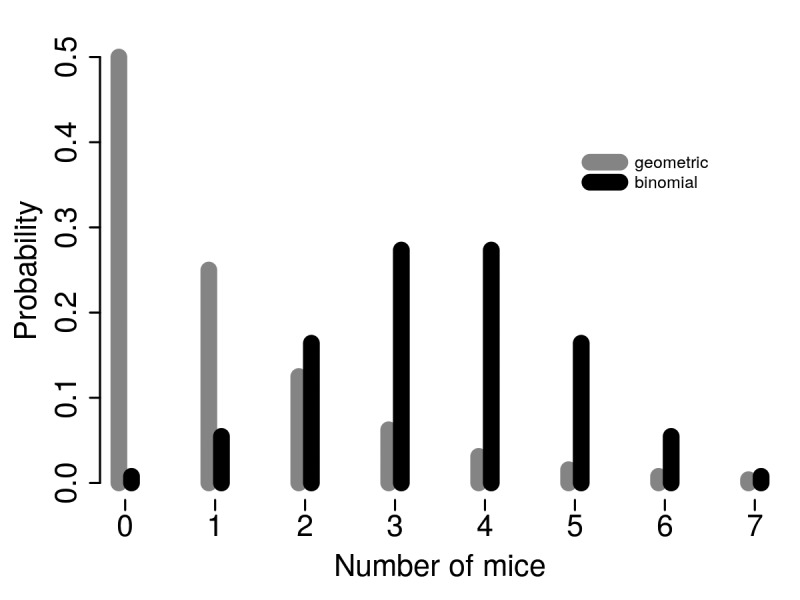
Two different experimental designs have different null distributions and consequently could lead to different interpretation of the same outcome. Gray – geometric; black – binomial.

**Figure 3:**
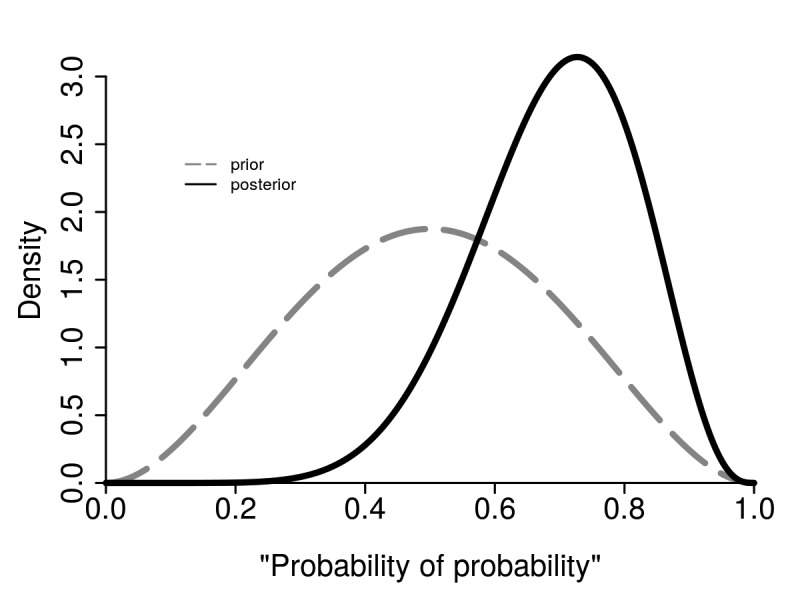
In Bayesian statistics it is not important which experimental design was applied as it is based on prior and posterior distribution functions. Gray – prior; black – posterior.

Another very important feature of frequentist statistics is that experimental design must be specified in advance. This difference between frequentist and Bayesian inference can be illustrated with the following example. Let us suppose that we want to investigate whether the sex ratio in hypothetical mice population is 1:1. We can create two experimental designs. In the first experiment, we can randomly select a mouse until the first male is chosen. The result in this experiment is the total number of mice chosen. In the second experiment, we can randomly select exactly seven mice. The result of this experiment will be the number of male and female mice in a sample of seven. Let us suppose that the result was FFFFFFM. If we do not know what experimental design was used, this result is useless. In the first experiment, the *P* is 0.031, but in the second experiment, the *P* value is 0.227. According to the common practice and the usual level of significance (0.05), we have to make two opposite conclusions from the same data. The origin of this difference is in the different null distributions, as the first was geometrical and the second was binomial (Figure 2). If we use Bayesian statistics, it does not matter which experimental design was applied. In Bayesian statistics a very common function used as a prior is Beta. If we choose Beta [3,3] function as a prior, the posterior function, according to the obtained result, will be Beta [9,4]. We can understand the Beta function as a function of the probability of the occurrence of specific parameters. Like other distribution functions, the Beta function can have different shapes, but with the domain in the interval [0,1]. Therefore, it is possible to calculate the probability that the sex ratio in this mice population is not 1:1 with Beta function as a prior. In this case it would be *P* = 0.92, ie, 92%, regardless of the experimental design (Figure 3).

A good example of the advantages of Bayesian statistics is the comparison of two data sets. Classical statistical procedures are F-test for testing the equality of variances and *t* test for testing the equality of means of two groups of outcomes. Both these tests are meaningful only if we can prove the normal distribution of the hypothetical population from which the samples originated (in fact, we will estimate the distribution of values never measured). Whatever method of frequentist statistics we use, the null hypothesis is always that the samples come from the same population (that there is no statistically significant difference in the parameters tested between samples). Since the distribution function of parameters is known (t-distribution, F-distribution, etc), it is easy to calculate how large the appropriate statistics must be in order to have a *P* value that is lower or equal to the desired *P* value (it is the so-called limit value of the test statistics for the desired significance level) (2).

In other words, frequentist statistics estimates the desired confidence percentage (usually 95%) in which some parameter is placed. In contrast, Bayesian analysis answers the following question: “What is the probability of the hypothesis given the measured data?” In addition, frequentist statistics accepts or rejects the null hypotheses, but Bayesian statistics estimates the ratio of probabilities of two different hypotheses. This ratio is known as the Bayesian ratio or Bayesian factor (3) (Table 1). The Bayesian factor quantifies the support for one hypothesis over another, regardless of whether these hypotheses are correct.

**Table 1:** The meaning of the Bayes factors

Bayes factor	Meaning
>100	Extreme evidence for H1
30-100	Very strong evidence for H1
10-30	Strong evidence for H1
3-10	Moderate evidence for H1
1-3	Anecdotal evidence for H1
1	No evidence
1/3-1	Anecdotal evidence for H0
1/3-1/10	Moderate evidence for H0
1/10-1/30	Strong evidence for H0
1/30-1/100	Very strong evidence for H0
<1/100	Extreme evidence for H0

There are many advantages and disadvantages of both frequentist and Bayesian statistics. Frequentist statistics never uses or calculates the probability of the hypothesis, while Bayesian uses probabilities of data and probabilities of both hypothesis. Frequentist methods do not demand construction of a prior and depend on the probabilities of observed and unobserved data. On the other hand, Bayesian methods depend on a prior and on the probability of the observed data (4).

There are more and more claims that Bayesian statistics is much more convenient for clinical research (5), and more attempts of using both frequentist and Bayesian statistics for data processing in clinical research, but the importance of Bayesian statistics also increases because it is fundamental for machine learning algorithms, ie, systems based on artificial intelligence (6). It is hard to be a judge and unequivocally “support” one of these statistics. However, judging is not even necessary. Forcing to use only one kind of statistics would be equal to forcing the use of laser scalpel over a regular one when it is known that in certain procedures the advantage of one is a disadvantage of the other. Hence, we should understand the Bayesian statistics as another powerful tool to process our data.
